# Energy-dense, low-volume paediatric oral nutritional supplements improve total nutrient intake and increase growth in paediatric patients requiring nutritional support: results of a randomised controlled pilot trial

**DOI:** 10.1007/s00431-020-03620-9

**Published:** 2020-03-13

**Authors:** Gary P. Hubbard, Catherine Fry, Katy Sorensen, Catherine Casewell, Lydia Collins, Annaruby Cunjamalay, Michelle Simpson, Amanda Wall, Elmarie Van Wyk, Matthew Ward, Sophie Hallowes, Hannah Duggan, Jennifer Robison, Helen Gane, Lucy Pope, Jennifer Clark, Rebecca J. Stratton

**Affiliations:** 1 Nutricia Ltd, Medical Affairs, Trowbridge, UK; 2 Department of Nutrition and Dietetics, Ashford and St Peter’s Hospitals NHS Foundation Trust, Chertsey, UK; 3 Department of Nutrition and Dietetics, Cumbria Partnership NHS Foundation Trust, Whitehaven, UK; 4 Department of Nutrition and Dietetics, Hampshire Hospitals NHS Foundation Trust, Winchester, UK; 5 Department of Nutrition and Dietetics, East Lancashire Hospitals NHS Trust, Blackburn, UK; 6 Department of Nutrition and Dietetics, Great Western Hospitals NHS Foundation Trust, Swindon, UK; 7 Department of Nutrition and Dietetics, Cwm Taf University Health Board, Prince Charles Hospital, Merthyr Tydfil, UK; 8 Department of Nutrition and Dietetics, Guy’s and St Thomas’ NHS Trust, London, UK; 9 Department of Nutrition and Dietetics, Lewisham and Greenwich NHS Trust, London, UK; 10 Department of Nutrition and Dietetics, Western Sussex Hospitals NHS Foundation Trust, Chichester, UK; 11 Department of Nutrition and Dietetics, Airedale NHS Foundation Trust, Keighley, UK; 12 Faculty of Medicine, University of Southampton, Southampton, UK

**Keywords:** Paediatric, Growth, Faltering, Oral, Nutrition, Intake

## Abstract

Children with or at risk of faltering growth require nutritional support and are often prescribed oral nutritional supplements (ONS). This randomised controlled trial investigated the effects of energy-dense paediatric ONS (2.4 kcal/ml, 125 ml: cONS) versus 1.5 kcal/ml, 200 ml ONS (sONS) in community-based paediatric patients requiring oral nutritional support. Fifty-one patients (mean age 5.8 years (SD 3)) with faltering growth and/or requiring ONS to meet their nutritional requirements were randomised to cONS (*n* = 27) or sONS (*n* = 24) for 28 days. Nutrient intake, growth, ONS compliance and acceptability, appetite and gastro-intestinal tolerance were assessed. Use of the cONS resulted in significantly greater mean total daily energy (+ 531 kcal/day), protein (+ 10.1 g/day) and key micronutrient intakes compared with the sONS group at day 28 and over time, due to high ONS compliance (81% of patients ≥ 75%), maintained intake from diet alone and improved appetite in the cONS group, compared with the sONS group. Although growth increased in both intervention groups, results were significant in the cONS group (weight (*p* = 0.007), height (*p* < 0.001) and height *z*-score (*p* = 0.006)).

*Conclusions*: This study shows that use of energy-dense (2.4 kcal/ml) low-volume paediatric-specific ONS leads to improved nutrient intakes, growth and appetite in paediatric patients requiring oral nutrition support compared with standard energy density ONS.

*Trial registration*: The trial is registered at clinicaltrials.gov, identification number NCT02419599.

**Table:** 

**What is Known:** • *Faltering growth is the failure of children to achieve adequate growth at a normal rate for their age and requires nutritional support, including the use of oral nutritional supplements (ONS).* • *Energy-dense, low-volume ONS have benefits over standard ONS in adults.*	
**What is New:** • *This is the first RCT to investigate the effects of energy-dense, low-volume ONS (2.4 kcal/ml, 125 ml) in children with faltering growth, showing significant improvements in total nutrient intake and increased growth.* • *Energy-dense, low-volume ONS can play a key role in the management of faltering growth.*

**What is Known:**

• *Faltering growth is the failure of children to achieve adequate growth at a normal rate for their age and requires nutritional support, including the use of oral nutritional supplements (ONS).*

• *Energy-dense, low-volume ONS have benefits over standard ONS in adults.*

**What is New:**

• *This is the first RCT to investigate the effects of energy-dense, low-volume ONS (2.4 kcal/ml, 125 ml) in children with faltering growth, showing significant improvements in total nutrient intake and increased growth.*

• *Energy-dense, low-volume ONS can play a key role in the management of faltering growth.*

## Introduction

Faltering growth is the failure of children to achieve adequate growth at a normal rate for their age, as a result of inadequate nutritional intake/absorption of nutrients in relation to their requirements. Effective management through nutritional support is important for physical growth and development [19, 35, 53], and strategies aim to increase nutrient intake and promote ‘catch-up’ growth [23]. Where faltering growth continues and dietary intake alone is insufficient, multi-nutrient, nutritionally complete oral nutritional supplements (ONS) specifically designed for children should be considered to help patients meet their nutritional requirements [21]. These have been shown to be effective at improving nutrient intakes, growth and outcomes [1, 4, 12, 15, 20, 24, 33, 34, 36, 43] (reviewed [49] and included in guidelines [25, 31]), although evidence is often in specific patients groups including Crohn’s disease, cystic fibrosis and cancer.

Ready-made, liquid ONS designed for children and available on prescription typically provide 1–1.5 kcal/ml in 200ml bottles. However, paediatric patients with increased energy requirements, fluid restriction, poor feed tolerance and/or appetite loss due to the effect of disease and its treatment, may struggle to achieve their nutritional requirements with currently available options. One possible strategy to improve nutrient intake in children with faltering growth is to reduce the volume of ONS by increasing the energy and nutrient-density, which when undertaken with food has been shown to increase energy intake and appetite without affecting fullness [3, 8, 22, 28–30]. Indeed, a positive correlation between ONS compliance and ONS energy-density has been shown [18] and studies in adults have shown that energy-dense (2.4 kcal/ml), low-volume (125 ml) multi-nutrient ready-made liquid ONS (or ‘compact-style’) significantly increase compliance, total energy and protein intakes, body weight, and play a key role in oral nutrition support strategies for adult disease-related malnutrition in clinical practice in the EU and other countries [7, 13, 16, 17, 38, 39, 41, 42, 51]. Whilst it can be hypothesised that a similar effect would be seen with energy-dense, low-volume ONS in children, no comparative studies have been published to date.

This pilot trial aimed to investigate the effect of a paediatric-specific compact-style ONS on nutrient intake and growth in paediatric patients requiring nutritional support, over 28 days.

## Materials and methods

Community-based paediatric patients (≥ 1 year and < 12 years) with faltering growth and/or requiring ONS to meet their nutritional requirements, were recruited between August 2015 and March 2016. Exclusion criteria were major hepatic/renal dysfunction; galactosaemia/severe lactose intolerance; requirement of total enteral tube/parenteral or elemental/semi-elemental feeding; participation in other recent clinical studies; or investigator concern to comply with the protocol.

The study was a prospective, interventional, parallel, randomised controlled trial undertaken in *n* = 11 UK healthcare centres. Randomisation used codes generated from 10-block random number tables [27], and sealed, opaque envelopes with sequential number labels. Patients were randomised to receive either an energy-dense, low-volume paediatric-specific ready-made, liquid ‘compact-style’ ONS (cONS) (Fortini Compact Multi Fibre®, Nutricia: 2.4 kcal/ml, 300 kcal/125ml bottle, Table 1) or a 1.5 kcal/ml ONS (sONS) (any 1.5 kcal/ml, 300 kcal/200ml bottle, multi-nutrient, liquid paediatric ONS for children aged ≥ 1 year, Table 1) to be taken orally every day for 28 days (volume determined by Dietitian according to local protocols and clinical judgement), in addition to appropriate nutritional management. Blinding to group allocation was not possible due to the different sizes of the ONS bottles.

**Table 1:** Nutritional composition of the energy-dense, low-volume oral nutritional supplement (cONS, Fortini Compact Multi Fibre®, Nutricia)^a^ and the standard ONS control feeds used (sONS)^b, c^

Nutritional composition	cONS per 125-ml bottle	sONS per 200-ml bottle
Energy, kcal	300	300
Energy density, kcal/ml	2.4 kcal/ml	1.5 kcal/ml
Protein, g (% En)	7.1 (10%)	6.6–8.4 (9–11%)
Carbohydrate, g (% En)	35.6 (47%)	33.4–37.6 (44–50%)
Fat, g (% En)	13.6 (41%)	13.6–14.9 (40–45%)
Fibre, g (% En)	3.0 (2%)	0–3.0 (0–2%)
Osmolality, mOsmol/kg H_2_O	975	350–595

^a^Fortini Compact Multi Fibre also contains a full range of vitamins and minerals with nutritionally complete volumes of 899 ml for 1–3 years, 1204 ml for 4–6 years and 2022 ml for 7–10 years [9]

^b^sONS group received Fortini Multi Fibre®/Nutricia, Fortini®/Nutricia or Paediasure Plus®/Abbott. Data presented are ranges for the 3 ONS used

^c^Macronutrient sources: protein, cow’s milk; fat, vegetable oils; carbohydrate, maltodextrin, glucose, sucrose

The primary outcome of nutrient intake using 24-h dietary recall was recorded at baseline and day 28 (Nutritics Professional v3.09, Nutritics, Ireland), nutrient intake from ONS alone was calculated from ONS compliance, which was recorded daily, and percentage of patients with a mean ONS compliance ≥ 75% calculated. The Dietitian’s expectation of ONS compliance was recorded. Study ONS acceptability was recorded at day 28 using 7-point Likert scales for pleasantness, enjoyment of taste and thickness (I dislike it very much–I like it very much). Growth outcomes (weight/kg (for children < 2 years: calibrated infant weighing scale with tray (accurate to 0.01–0.02 kg); for children > 2 years: calibrated electronic weighing scale or a beam balance. Up to 10 kg accurate to 10 g; up to 20 kg accurate to ± 20 g and over 20 kg accurate to 50–200 g), height/cm (for children < 2 years old or children unable to stand without assistance: supine length; for children > 2 years old standing without assistance: standing height with stadiometer; for children > 2 years old unable to stand without assistance: extrapolated from ulna length or knee height), head circumference/cm in children ≤ 2 years using a slotted non-stretchable insertion tape accurate to 0.1 cm) were measured at baseline and day 28 using standardised methods. *Z*-scores were calculated using an online algorithm for weight and height [40]. Improvement in appetite was recorded at day 28 (improved; stayed the same; reduced). Gastrointestinal tolerance at baseline and day 28 (incidence and severity of gastrointestinal symptoms) and (serious) adverse events were recorded.

**Fig. 1:**
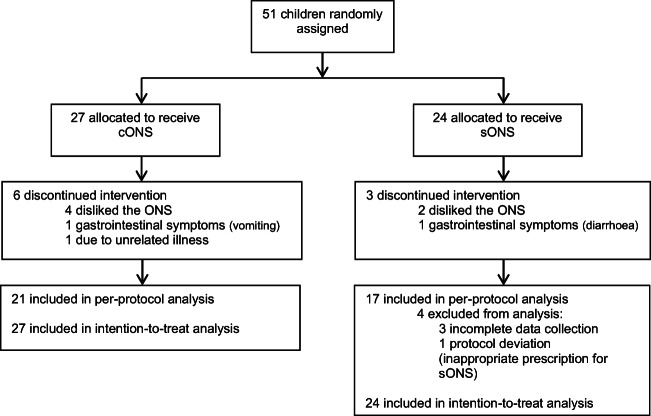
Flow chart of study exclusions and participation

This was a pilot study due to the lack of published data in this area; however, similar studies have used patient groups of 7–25 [11, 14, 15, 36, 43, 52]. A power calculation was conducted calculating that a sample size of 23 per group would be sufficient to detect a difference in total daily energy intake of 500 kcal (SD 600) between groups with 80% power and a *p* value of 0.05; therefore, a target sample size of 50 (25/group), allowing for dropouts, was deemed reasonable. Data were assessed for normal distribution and analysed as intention to treat (ITT: *n* = 51, all patients entering the trial), and per protocol (PP: *n* = 38, excluding dropouts and exclusions, Fig. 1), using IBM SPSS version24.0 (IBM SPSS Statistics, Aramonk, USA). Differences between groups in nutrient intake and growth parameters at day 28 were adjusted for baseline (and age, where appropriate) using univariate ANOVA and appropriate non-parametric testing. Mean total and individual micronutrient intakes from diet and ONS for patients within the age groups 1–3 years, 4–6 years, 7–10 years and 11–14 years were compared with UK-specific reference nutrient intakes (RNIs) [9]. Categorical data was analysed using Pearson’s chi-square test, Fisher’s exact test and Wilcoxon signed-rank test as appropriate. For the ITT analysis, multiple imputation [44] was used for nutrient intake, growth and compliance. For any single variable, missing data varied 3.9–9.8%. Five multiple-imputed datasets with 10 iterations using a linear regression model were created. Variables included were age at baseline, gender, compliance, mean volume of ONS consumed/day, energy requirements/day at baseline, and baseline and day 28 data for all nutrient intake outcomes and anthropometric variables. Pooling of data was undertaken using Rubin’s rule [32], and the associated degrees of freedom and *p* values were calculated using the methodology recommended by Barnard and Rubin [2].

## Results

Fifty-one patients were randomised and eligible for ITT (cONS *n* = 27; sONS *n* = 24). Dropout was 22% with the cONS and 13% with the sONS (*p* = 0.297), with a further *n* = 4 exclusions (Fig. 1). Consequently, *n* = 38 were eligible for PP analysis (cONS *n* = 21; sONS *n* = 17, *p* = 0.402).

There were no significant differences between groups for all baseline patient characteristics (Table 2), apart from age in the PP analysis which was significantly higher with the cONS compared with the sONS (*p* = 0.039). Primary diagnosis was faltering growth in the majority of patients (*n* = 21) (mean *z*-scores for weight, height and head circumference indicating an approach towards faltering growth, or poor weight gain), with underlying conditions including respiratory (*n* = 11), genetic (*n* = 7), central nervous system (*n* = 7), gastro-intestinal (*n* = 5), cardiac (*n* = 4), neurodevelopmental (*n* = 4), prematurity (*n* = 3), intrauterine growth retardation (*n* = 2), developmental delay (*n* = 1), and autoimmune (*n* = 1) conditions. The patients had low weight and height for age, and total energy intakes below calculated requirements (Table 2). Most (71%, *n* = 36) were already prescribed an ONS at baseline. For the sONS *n* = 7 remained on the 1.5 kcal/ml ONS previously taken, whereas *n* = 17 changed to a different 1.5 kcal/ml ONS on entering the trial. The sONS group received Fortini Multi Fibre®/Nutricia, Fortini®/Nutricia or Paediasure Plus®/Abbott. Mean prescribed energy from the study ONS did not differ between groups (cONS 496 kcal/day (SD 165) versus sONS 467 kcal/day (SD 179), *p* = 0.561) with both groups prescribed a mean 1.6 bottles/day.

**Table 2:** Baseline characteristics by intervention group (ITT population, *n* = 51)

	cONS (*n* = 27)	sONS (*n* = 24)
Age	6 years 3 months (3 years 1 month)	5 year 3 months (3 years 7 months)
Male/female (*n*)	17/10	14/10
Total energy requirement, kcal/day	1682 (851)	1397 (467)
Total energy intake, kcal/day	1450 (654)	1266 (511)
Weight, kg	17.8 (7.2)	16.1 (8.0)
Weight *z*-score	− 2.11 (1.41)	− 2.15 (1.45)
Height, cm	108.5 (20.8)	101.3 (24.8)
Height *z*-score^a^	− 1.54 (1.23)	− 1.71 (1.51)
Head circumference, cm^b^	45.3 (0.4)	44.1 (1.1)
Head circumference *z*-score^c^	− 1.70 (0.42)	− 2.35 (0.21)

Data presented as mean (SD) or number of subjects. cONS (energy dense, low volume ONS); sONS (standard ONS)

^a^*n* = 49 as inappropriate to impute missing data (*n* = 2) for *z*-scores

^b^*n* = 6 as head circumference was only measured in children < 2 years of age

^c^*n* = 4 as inappropriate to impute missing data (*n* = 2) for *z*-scores

There were no significant differences between ITT intervention groups for all included baseline characteristics. For the PP analysis, there was a significant difference in age between intervention groups, *p* = 0.039, with mean age being higher in the cONS group

**Fig. 2:**
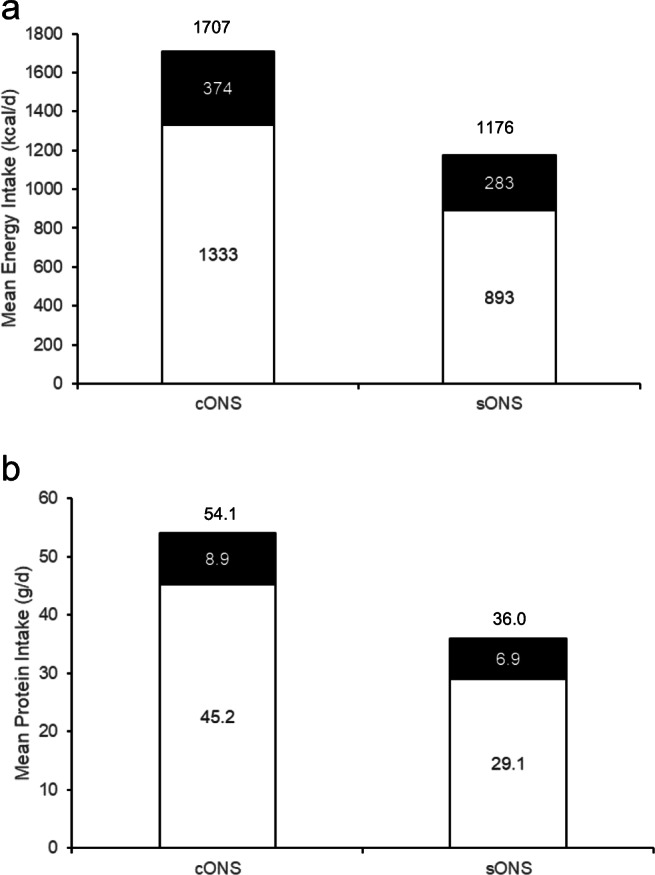
Mean daily energy (**a**) and protein (**b**) intakes are higher with cONS than with sONS at day 28 (intake from diet: white bar; intake from ONS: black bar. Total value provided above bar

Mean total daily energy intake from diet and ONS was significantly higher at day 28 with the cONS (1707 kcal/day, 95% CI 1458, 1956) versus the sONS (1176 kcal/day, 95%CI 889, 1453), a difference of + 531 kcal/day (PP: *p* = 0.008 unadjusted, *p* = 0.059 adjusted for baseline energy intake and age) (Fig. 2a and Table 3), and increased over time with the cONS (ITT + 132 kcal/day, 95%CI − 56, 320, *p* = 0.169; PP + 168 kcal/day, 95%CI − 61, 396, *p* = 0.151) but remained stable with the sONS. The percentage of patients meeting their calculated energy requirements increased with the cONS over time (33 to 48%, *NS*), but did not change with the sONS (33% and 33%, *NS*) (ITT and PP). Mean daily energy intake from diet alone was significantly greater with the cONS compared with the sONS at day 28 (*p* = 0.017 unadjusted, *p* = 0.159 adjusted for baseline energy intake) (Fig. 2a and Table 4) but did not change significantly over time in either group.

**Table 3:** Mean total daily nutrient intake from diet and study ONS at day 28 (ITT and PP analysis) for cONS and sONS intervention groups

	ITT analysis			PP analysis		
	cONS *n* = 27	sONS *n* = 24	*p* value	cONS *n* = 21	sONS *n* = 17	*p* value
Energy, kcal	1582 (1339, 1826)	1304 (1056, 1551)	0.303	1707 (1458, 1956)	1176 (889, 1453)	0.059
Protein, g	51.1 (43.7, 58.4)	41.0 (33.1, 48.8)	0.050	54.1 (46.6, 61.7)	36.0 (27.6, 44.4)	0.011
Fluid, ml	837 (698, 977)	768 (620, 916)	0.458	888 (727, 1048)	755 (576, 933)	0.613
Fibre, g	13.6 (11.4, 15.8)	8.6 (6.3, 11.0)	0.004	15.5 (13.1, 17.9)	8.4 (5.7, 11.0)	0.001
Sodium, mg	1388 (1100, 1676)	1280 (1035, 1526)	0.865	1510 (1207, 1812)	1148 (812, 1484)	0.320
Potassium, mg	2005 (1706, 2305)	1423 (1106, 1740)	0.025	2177 (1841, 2514)	1283 (909, 1656)	0.009
Phosphorus, mg	945 (806, 1085)	714 (567, 862)	0.009	1016 (879, 1152)	631 (479, 782)	0.002
Calcium, mg	812 (674, 951)	662 (515, 809)	0.116	860 (730, 991)	600 (456, 745)	0.021
Magnesium, mg	186 (159, 214)	140 (110, 169)	0.052	201 (170, 231)	130 (96, 163)	0.026
Iron, mg	10.3 (8.5, 12.1)	8.5 (6.5, 10.4)	0.161	10.9 (9.0, 12.7)	7.7 (5.7, 9.8)	0.052
Zinc, mg	9.0 (7.4, 10.6)	7.4 (5.7, 9.2)	0.104	9.7 (8.1, 11.2)	6.5 (4.7, 8.2)	0.014
Vitamin A, μg	661 (517, 806)	529 (576, 682)	0.261	670 (514, 827)	515 (340, 689)	0.220
Vitamin D, μg	5.9 (4.6, 7.2)	4.7 (3.2, 6.1)	0.072	674 (510, 839)	509 (325, 693)	0.009
Vitamin E, mg	10.4 (8.5, 12.4)	8.3 (6.2, 10.3)	0.249	11.1 (9.2, 13.1)	7.6 (5.4, 9.8)	0.056
Thiamin, mg	1.5 (1.3, 1.8)	1.3 (1.0, 1.6)	0.266	1.6 (1.3, 1.9)	1.2 (0.9, 1.5)	0.069
Riboflavin, mg	1.6 (1.3, 1.9)	1.5 (1.2, 1.8)	0.416	1.7 (1.4, 2.0)	1.4 (1.1, 1.7)	0.251
Niacin, mg	18.0 (14.8, 21.1)	16.5 (13.1, 19.8)	0.761	18.2 (14.6, 21.8)	14.2 (10.2, 18.1)	0.389
Vitamin B6, mg	1.5 (1.2, 1.7)	1.1 (0.9, 1.4)	0.062	1.5 (1.3, 1.8)	1.1 (0.8, 1.4)	0.026
Vitamin B12, μg	3.7 (3.0, 4.4)	2.9 (2.2, 3.6)	0.031	4.0 (3.3, 4.8)	2.6 (1.8, 3.4)	0.014
Folic acid, μg	141 (112, 171)	130 (98, 161)	0.934	134 (104, 165)	120 (85, 154)	0.976
Vitamin C, mg	81.6 (64.1, 99.8)	61.0 (42.4, 79.6)	0.081	83.0 (65.1, 101.0)	59.4 (39.4, 79.30	0.043

Values are mean (95%CI). *p* values (adjusted)

**Table 4:** Mean daily nutrient intake from diet alone at day 28 (ITT and PP analysis) for cONS and sONS intervention groups

	ITT analysis			PP analysis		
	cONS *n* = 27	sONS *n* = 24	*p* value	cONS *n* = 21	sONS *n* = 17	*p* value
Energy, kcal	1291 (1071, 1511)	1034 (805, 1264)	0.245	1333 (1103, 1562)	893 (638, 1149)	0.159
Protein, g	44.1 (37.2, 51.1)	34.7 (27.3, 42.1)	0.039	45.2 (38.2, 52.3)	29.1 (21.2, 37.0)	0.019
Fluid, ml	837 (701, 974)	604 (489, 779)	0.021	888 (734, 1041)	608 (438, 779)	0.075
Fibre, g	10.7 (8.8, 12.6)	7.2 (5.2, 9.3)	0.059	11.8 (9.5, 14.0)	6.6 (4.1, 9.1)	0.037
Sodium, mg	1280 (995, 1565)	1177 (875, 1479)	0.913	1371 (1069, 1672)	1044 (709, 1379)	0.394
Potassium, mg	1737 (1446, 2028)	1204 (907, 1502)	0.042	1832 (1491, 2174)	1062 (683, 1441)	0.049
Phosphorus, mg	760 (632, 887)	594 (459, 729)	0.045	777 (651, 904)	511 (371, 652)	0.035
Calcium, mg	609 (483, 734)	525 (392, 658)	0.375	598 (482, 714)	465 (336, 594)	0.265
Magnesium, mg	155 (130, 180)	111 (84, 138)	0.039	160 (131, 189)	101 (68, 133)	0.071
Iron, mg	7.4 (5.9, 8.9)	5.8 (4.1, 7.4)	0.048	7.1 (5.6, 8.7)	4.9 (3.2, 6.7)	0.179
Zinc, mg	6.1 (4.8, 7.4)	4.7 (3.3, 6.2)	0.030	5.9 (4.7, 7.2)	3.7 (2.3, 5.1)	0.049
Vitamin A, μg	534 (391, 676)	403 (252, 553)	0.250	506 (353, 660)	377 (207, 547)	0.289
Vitamin D, μg	2.1 (1.1, 3.2)	2.2 (1.1, 3.3)	0.607	1.5 (0.6, 2.3)	1.7 (0.8, 2.6)	0.284
Vitamin E, mg	6.7 (5.0, 8.3)	4.9 (3.2, 6.7)	0.388	6.3 (4.6, 8.0)	4.3 (2.4, 6.2)	0.591
Thiamin, mg	1.1 (0.9, 1.3)	0.9 (0.7, 1.2)	0.245	1.0 (0.8, 1.3)	0.8 (0.5, 1.0)	0.199
Riboflavin, mg	1.2 (0.9, 1.4)	1.1 (0.8, 1.3)	0.343	1.1 (0.9, 1.4)	0.9 (0.7, 1.2)	0.640
Niacin, mg	18.0 (14.8, 21.1)	13.4 (10.1, 16.8)	0.075	18.2 (14.7, 21.7)	10.9 (7.0, 14.8)	0.037
Vitamin B6, mg	1.1 (1.0, 1.2)	0.9 (0.6, 1.10	0.087	1.1 (0.9, 1.3)	0.8 (0.5, 1.0)	0.103
Vitamin B12, μg	3.2 (2.6, 3.9)	2.4 (1.7, 3.1)	0.033	3.4 (2.7, 4.1)	2.1 (1.3, 2.9)	0.020
Folic acid, μg	141 (113, 170)	65 (65, 125)	0.034	134 (2.7, 4.1)	82 (49, 116)	0.094
Vitamin C, mg	52.5 (36.8, 68.2)	36.8 (20.0, 53.5)	0.181	45.6 (30.2, 61.0)	355 (18.4, 52.6)	0.390

Values are mean (95%CI). *p* values (adjusted)

Mean total daily protein intake from diet and ONS was significantly higher at day 28 with the cONS (51.1 g/day, 95% CI 43.7, 58.4) versus the sONS (41.0 g/day, 95%CI 33.1, 48.8, ITT), a difference of + 10.1 g/day (*p* = 0.050, adjusted for baseline total protein intake; PP: *p* = 0.011, Fig. 2b and Table 3), and increased over time with the cONS (ITT +3.2 g/day, 95%CI − 2.2, 8.7, *p* = 0.246; PP + 3.1 g/day, 95%CI − 3.8, 10.0, *p* = 0.376), although there was no change with the sONS. Mean daily protein intake from diet alone was significantly higher with the cONS compared with the sONS at day 28 in both ITT and PP (*p* = 0.039 and *p* = 0.019 respectively, adjusted for mean baseline protein intake) (Fig. 2b and Table 4), but did not change significantly over time with the cONS (ITT + 2 g/day, 95%CI − 4.2, 8.2; PP − 0.07 g/day, 95%CI − 7.6, 7.5), and decreased slightly over time with the sONS (ITT − 5.0 g/day, 95%CI − 10.3, 0.3, *NS*; PP − 6.1 g/day, 95%CI − 11.7, − 0.5, *p =* 0.033). There were no statistically significant differences in mean total daily fluid intake from diet and ONS between groups at day 28, or changes over time (Table 3). Mean daily fluid intake from diet alone (i.e. from sources other than the study ONS) increased with the cONS over time (ITT + 146 ml/day, 95%CI 22, 270, *p =* 0.021; PP + 121 ml/day, 95%CI − 33, 275, *NS*), but did not change with the sONS (ITT − 47 ml/day, 95%CI − 179, 84, *NS*; PP + 1.8 ml/day, 95%CI − 134, 137, *NS*). Mean total daily micronutrient intakes from diet and ONS at day 28 were significantly greater with the cONS compared with the sONS for potassium, phosphorus and vitamin B12 in the ITT (adjusted for baseline values), and for potassium, phosphorus, calcium, magnesium, zinc, vitamin D, vitamin B6, vitamin B12 and vitamin C in the PP (Table 3), and also increased over time with the cONS (significant increases of phosphorus, iron, zinc, vitamin D, thiamin, riboflavin, vitamin B6, vitamin B12 and vitamin C, *p* < 0.05); however, with the sONS only the vitamin D intake increased significantly over time (ITT *p* = 0.035), with similar results for the PP. At baseline there were 9/17 micronutrients for which the mean total daily intake was below the UK RNI in at least 1 age group with the cONS, which improved to 3/17 micronutrients at day 28 (ITT), with similar results for the PP (baseline 9/17 vs. day 28 2/17) (*NS*), which were not seen with the sONS (baseline 8/17 vs. day 28 10/17, *NS*, ITT). Mean daily micronutrient intakes from diet alone were significantly greater with the cONS compared with the sONS at day 28 for potassium, phosphorus, magnesium, zinc, vitamin B12 and folic acid (ITT, Table 4), with similar results for the PP, and significant increases over time with the cONS (zinc, thiamin, riboflavin, vitamin B6 and vitamin B12, *p <* 0.05, ITT) that were not observed with the sONS over time.

Mean daily energy and protein intakes from the ONS alone were higher with the cONS compared with the sONS throughout the trial (ITT: cONS 352 kcal/day, 95%CI 287, 417 and 8.3 g/day protein, 95%CI 6.7, 10.0 vs. sONS 326 kcal/day, 95%CI 257, 395 and 7.9 g/day protein, 95%CI 6.2, 9.6), although there were no significant differences between groups.

With the cONS there were a significantly higher number of patients consuming ≥ 75% of their prescribed ONS versus those consuming less (81% vs. 19%, *p* = 0.005, PP), which was not apparent with the sONS (59% vs. 41%, *p* = 0.467, PP). The percentage of patients who met or exceeded their Dietitian’s expectations for ONS compliance was higher with the cONS (86%) compared with the sONS (71%) (*p* = 0.426, PP). The cONS was well accepted with 60% finding it pleasant to drink, 65% enjoying the taste and 68% liking the thickness.

Significant increases in weight (*p* = 0.007), height (*p* < 0.001) and height *z*-score (*p* = 0.006) were observed over time with the cONS, which were not apparent with the sONS (Table 5). There were no significant differences between groups in mean weight (cONS 19.6 kg (SD 6.9) vs. sONS 15.5 kg (SD 8.1), Δ4.1 kg, PP) at day 28 or weight *z*-score (cONS − 1.79 (SD 1.21) vs. sONS − 1.88 (SD 1.16), Δ0.09, PP) at day 28 (PP, adjusted for baseline). However, mean height (cONS 114.4 cm (SD 18.2) vs. sONS 99.3 cm (SD 24.4) Δ15.1 cm, PP) and height *z*-scores (cONS − 1.25 (SD1.19) vs. sONS − 1.55 (SD 0.99), Δ0.3, PP) were significantly higher with the cONS versus the sONS at day 28 (*p* < 0.001 and *p* = 0.004, respectively, PP adjusted for baseline). With the cONS a significantly greater proportion of patients reported their appetite had improved over time (48%), compared with the sONS (12%, *p* = 0.018).

**Table 5:** Improvements in growth in both intervention groups from baseline (BL) to day 28 (PP analysis)

	cONS, *n* = 21		sONS, *n* = 17	
	Change from BL to day 28	*p* value	Change from BL to day 28	*p* value
Weight (kg)	0.28 (0.08, 0.47)	0.007	0.29 (− 0.05, 0.63)	0.094
Weight *z*-score	0.07 (− 0.01, 0.16)	0.083	0.07 (− 0.11, 0.25)	0.429
Height (cm)	0.87 (0.59, 1.16)	< 0.001	0.55 (0.17, 0.93)^a^	0.007
Height *z*-score	0.10 (0.03, 0.16)	0.006	− 0.02 (− 0.11, 0.06)^a^	0.569

Values are mean (95% CI); ^a^*n* = 16, missing data

Nine adverse events were reported, six with the cONS (2 patients) and three with the sONS (2 patients). Of these, four were deemed not related to the study product (cONS *n* = 2, sONS *n* = 2), three were gastrointestinal symptoms possibly related to the study product (cONS *n* = 2, sONS *n* = 1) and two were vomiting in the same patient highly probably related to the study product (cONS). The number of patients reporting gastrointestinal symptoms was low in both groups with no significant changes over time within groups.

## Discussion

This is the first study to investigate the effects of energy-dense, low-volume, paediatric ONS versus standard paediatric ONS on patient’s nutrient intake and growth. In both the ITT and PP analyses, the provision of a cONS in addition to appropriate nutrition support for 28 days led to significantly improved total nutrient intakes, greater nutrient intakes from diet alone, high compliance to the cONS, significant increases in growth and improved appetite versus the provision of standard 1.5 kcal/ml ONS.

The paediatric patients in this trial had a variety of conditions, but represent a typical faltering growth population, with low weight and height for age and energy intakes below calculated requirements, despite the majority being previously managed for faltering growth and receiving ONS. Effective management of faltering growth requires the provision of optimal energy, protein and micronutrients important for growth [21], and nutrition support strategies can include dietary advice, food fortification and the use of ONS. There is significant evidence supporting the use of ONS, particularly in adults [5–7, 10, 13, 17, 18, 26, 38, 39, 41, 42, 45, 47–51], but also in children [1, 4, 12, 15, 19–21, 24, 25, 31, 33, 34, 36, 43, 49, 53], showing improved nutrient intakes, weight, quality of life, and reduced hospital admissions and readmissions, complications and healthcare costs. However, there is a lack of evidence for the use of ONS in the general faltering growth population and for the use of ready-made (liquid) ONS. This trial shows that the use of a ready-made, energy-dense, low-volume ONS can improve nutrient intakes, increasing total energy intakes to meet calculated requirements, and increasing micronutrient intakes to meet age appropriate reference nutrient intakes, a key benefit of the multi-nutrient format of ONS. Furthermore, nutrient intake from diet alone was not negatively impacted by cONS consumption, which also improved patients’ appetite. These benefits are likely to be due to the energy-dense, low-volume (2.4 kcal/ml, 125 ml) nature of the cONS, having little impact on appetite, fullness and satiety. High compliance was also observed with the cONS, similar to that reported in studies of cONS in adults [13, 17], and higher than previously reported in children [37], most likely due to the good acceptability, and higher energy and nutrient density of the cONS in a smaller volume. In turn, this may allow a greater capacity to consume more foods. These findings are contrary to the suggestion that high-energy liquid feed supplements may suppress appetite and replace normal diet [25]. Importantly, use of both ONS led to growth with gains in both weight and height, which is consistent with the findings of a systematic review of 11 studies of improved growth in 220 patients aged 4 months–19 years with growth retardation and conditions including cystic fibrosis and Crohn’s disease, following the use of ONS [46]. Indeed, the growth observed in both groups is especially surprising over such a short period of only 28 days intervention.

This study has limitations. As this was a multi-centre pilot study, the results need to be interpreted with caution. Although the sample size was relatively low (indicative of difficult recruitment in this complicated and vulnerable patient group), it was comparable to or higher than other similar studies of nutritional support in paediatric patients with faltering growth [11, 14, 15, 36, 43, 52] and large enough to show significant benefits between interventions, in favour of the cONS. This trial aimed to compare the two ONS interventions with the standard ONS as the control group, and both groups included appropriate nutritional support, which was left to the Dietitians discretion and could have included dietary advice and/or food fortification. Other trial design options could include a control arm assessing dietary advice/food fortification, but that was not the research objective here. The patient group in the study were heterogenous in age and disease/condition, but typical of a faltering growth population requiring oral nutritional support in clinical practice. Due to the difference in size of the two study ONS bottles, blinding to the interventions was not possible. A small number of children who were randomised to the sONS were already consuming an ONS, and they continued on this throughout the study; however, there was no significant difference in compliance between children who continued with their previous ONS and those who switched to a different ONS. It should be noted that a relatively small but similar number of patients dropped out of both groups of the study due to gastrointestinal symptoms and disliking the taste of the ONS. The study did not assess the long-term effects of ONS usage beyond 4 weeks. Longer-term studies are warranted to assess the continuing effects of energy-dense, low-volume ONS.

In conclusion, use of energy-dense, low-volume paediatric-specific ONS in addition to appropriate nutritional management in paediatric patients with faltering growth in this pilot study, led to significantly improved nutrient intake with no impact on intake from diet, and significantly increased growth over 4 weeks, accompanied by high ONS compliance and improved appetite. Energy-dense, low-volume paediatric-specific ONS are therefore an effective alternative to standard paediatric ONS for children. Furthermore, this study adds to the evidence base for the use of ready-made ONS in children with faltering growth.
